# Allergic Contact Dermatitis Reaction to Permanent Tattoo Containing Paraphenylenediamine: A Case Report

**DOI:** 10.1155/2024/2118096

**Published:** 2024-09-25

**Authors:** Eliot Parascandolo, Samuel Puglisi, Miguel Marenco, Gregory Puglisi

**Affiliations:** ^1^ NCH Healthcare System Internal Medicine Department, Naples, Florida, USA; ^2^ University of Maryland College of Computer, Mathematical and Natural Sciences, College Park, Maryland, USA; ^3^ The National Autonomous University of Nicaragua, Managua, Nicaragua; ^4^ Mid-Island Allergy Group, Plainview, New York, USA

**Keywords:** allergic contact dermatitis, paraphenylenediamine, tattoo

## Abstract

Paraphenylenediamine (PPD) is a well-known culprit allergen in the literature and clinical practice. Although this has been described in temporary tattoos, the definite implication of PPD in permanent tattoos has not been described. We report a patient who developed severe allergic contact dermatitis (ACD) requiring skin grafting after receiving a permanent tattoo with ink containing PPD. A 30-year-old female with a past history of atopic dermatitis and psoriasis presented with a 2-week history of cutaneous reaction to a recent tattoo. The patient noticed inflammation and irritation of the tattoo site the day after administration. The patient was previously identified on patch testing to have a PPD allergy after evaluation for dermatitis after hair dye application. Following the tattoo placement, she applied soap and bacitracin cream which she had used several years prior on a similar tattoo. On presentation 2 weeks later, she was found to have a deep ulcerated plaque with an indurated border encompassing the area of the tattoo. She was referred to the emergency department and admitted for treatment, ultimately requiring debridement and skin grafting. The patient obtained the safety data sheets for the tattoo inks which revealed PPD as an ingredient in every color. We believe this is the first confirmed case of PPD being implicated as the causative agent for ACD to a permanent tattoo. Tattoo ink is unregulated, and formulas are proprietary which makes safe practice difficult for patients with sensitivities. We advocate for consistent ingredient labeling, regulation, and transparency within the tattoo ink industry.

## 1. Introduction

Tattoos are an ancient form of personal artistic expression that spans cultures. The practice of tattooing dates back to Japan 5000 BCE where ink compounds were primarily created from blackened soot and indigo [1]. Modern tattoos are performed using a tattoo gun which deposits a variety of ink substrates into the dermis.

Allergic contact dermatitis (ACD) to permanent tattoo is rarely reported in the literature; however, as tattoos become increasingly common, the incidence of ink associated reaction will likely increase as well. Data from an Ipsos poll revealed that tattoo prevalence has increased 21% between 2012 and 2019 [2]. Various studies demonstrate a 2–30% rate of cutaneous reactions to tattoo, and less than 1% of reactions are defined as ACD [3, 4]. Reactions to permanent tattoos can differ based on the constituents of ink, these reactions may be difficult to differentiate from infection and can also include granulomatous-type reactions.

ACD and photoallergic dermatitis are the most common hypersensitivity reactions against tattoo ink [5]. Unfortunately, ink production companies are not required to list ink ingredients which makes due diligence difficult for patients with sensitivities. Many brands' “ingredient lists” are difficult to produce, and simply list “proprietary blend.” Compounding this problem is the fact that many tattoo shops are unwilling to disclose the brand of ink to their clients. Many inks have similar base ingredients: isopropyl alcohol, glycerin, and hamamelis virginiana [6–8]. ACD to these constituents has all been previously documented [9–11]. ACD to a variety of common ink pigments, various metals, and adulterants including benzophenone again is reported in the data [12, 13].

P-Phenylenediamine is a common contact allergen. It has been the 10th most prevalent contact allergen in patch testing and has been voted the Allergen of the Year by the American Contact Dermatitis Society in 2006 [14, 15]. Although black henna and hair dye have been heavily implicated in PPD allergy, there is speculation of PPD use in permanent tattoo ink preparations causing reaction. Despite the prevalence of permanent tattoos, PPD reaction in the literature is exceedingly sparse. There is a report of a suspected reaction to PPD after a black ink tattoo; however, this reaction was defined as a 20-year history of pruritus and dermatologic changes to the tattoo area. The presence of PPD was also never confirmed [16]. In one study which included 38,543 individuals with tattoo reactions listed in the North American Contact Dermatitis Group, 29 patients had a diagnosis of tattoo-related ACD. Of these, 55% were found to have history of atopy. Screening series demonstrated PPD, cobalt, balsam of Peru, and nickel sulfate reactions. Of the 22 patients with documented PPD reaction, 50% were documented as extreme reactions including bullous and ulcerative reactions. In this study, however, only one participant had a “definite reaction” as defined in the study as a reaction where a patch test was verified to contain PPD. Two of the reactions were documented as probable, meaning the tattoo was confirmed to contain PPD, but patch testing was not performed [4]. It is important to note that this review included henna ink as well in which certain black henna inks are well-known to contain PPD.

Temporal reaction to PPD depends on prior exposure. If there has been prior sensitization, the time-to-reaction is between 1 and 3 days. However, if there has not been prior exposure, time-to-reaction can be as long as 14 days [17, 18]. As with most ACD patients, reactions can include edema, rubor, and erythema, vesicles which can progress to bullae [16]. One study noted that nearly a fourth of tattoo inks identified were composed of known contact allergens [19].

## 2. Case Presentation

We present a 30-year-old Caucasian female with a past medical history of atopic dermatitis, psoriasis, and ACD to black hair dye who presented for evaluation of cutaneous reaction at the site of a recent multicolored tattoo. The patient was previously being treated for atopic dermatitis affecting her eyelids, extremities, and trunk which had been difficult to control, and an evaluation with skin testing to environmental allergens was negative at that time. Patch testing with Smart Practice AC-Core series was placed shortly after the patient presented with scapular dermatitis which occurred following hair dye application. Her patch test was positive for nickel (+1), cobalt (+1), and p-phenylenediamine (+2).

**Figure 1:**
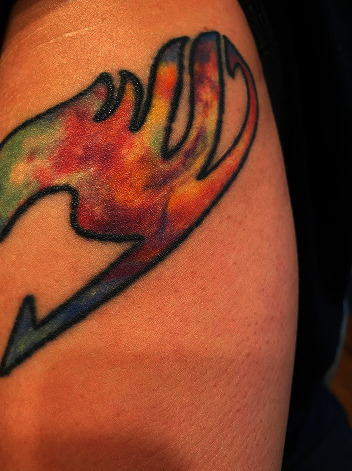
Prior multicolor tattoo without reaction.

The patient had the tattoo work done 2 weeks prior to her visit and noted an initial reaction of inflammation including redness and irritation approximately 1 day after the tattoo was applied. She had been cleaning the tattoo with “Hustle Bubble Tattoo Soap” and bacitracin, both of which she had used before without issue for a previous multicolored tattoo. Her prior multicolor tattoo, applied on her upper arm by the same artist, had no cutaneous reaction, but a different brand of tattoo ink was used as seen in Figure 1.

**Figure 2:**
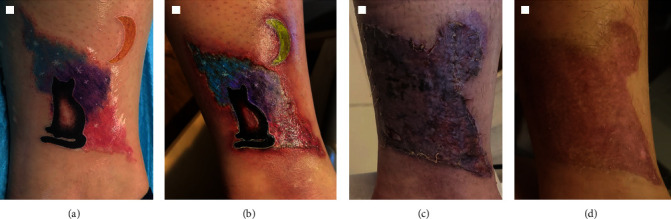
(a–d) Progression of tattoo reaction, subsequent skin grafting, and healing: (a) initial tattoo, (b) tattoo on evaluation demonstrating deep ulcerated plaque with indurated tattoo borders and erythema, (c) wound after debridement and skin grafting, and (d) healed wound demonstrating hyperpigmentation.

On presentation, she was found to have a deep ulcerated plaque with an indurated border encompassing the area of the tattoo. Given the severity of the reaction, the patient was referred to the emergency department where she underwent treatment with oral and topical steroids as well as antibiotics. The patient ultimately required debridement and skin grafting with resolution and healing of her wound. The progression of the reaction and healing is demonstrated in Figure 2a–d. Upon follow up, the patient was able to obtain the safety data sheets for the inks used in her tattoo which revealed PPD as an ingredient of every color. The skin graft healed with a remaining faint hyperpigmented lesion and no further reaction at the site of the tattoo.

## 3. Discussion

We present a patient with a severe cutaneous reaction at the site of a new tattoo that appears to be allergic contact in nature. She had already had reactions to black hair dye in the past and had tested positive for PPD on a previous patch test. This reaction would not be unusual for a temporary tattoo, such as a black henna tattoo which is known to contain PPD; however, it is unexpected for permanent tattoo inks to contain this agent. As tattoos become increasingly common, the incidence of ink associated reaction increases as well. For this reason, it is important to understand how these reactions occur and to consider ACD in the differential diagnosis in patients who present with signs of reaction after receiving a tattoo. Patients with allergic contact sensitization should be encouraged to obtain ingredient lists and tattoo ink names prior to tattooing. While most dermatologic cosmetic products are required to have labeled ingredients, tattoo inks, which are products that enter the dermis via needle and interact with the immune system, do not have this requirement. Compounding this fact is that tattoo ink manufacturers are unregulated with regard to performing tests for presence of adulterants. In addition to reporting this, the FDA also notes that microbiological contamination occurs often, making it challenging to distinguish between developing cellulitis and acute reactions [20]. Given this fact, it is imperative that the tattoo ink industry is held to higher standards including accurate and available ingredient lists, aseptic technique in ink production, and third-party testing to detect heavy metal and chemical adulterants. Furthermore, tattoo artists and shops should be transparent with the ink brand that will be used. Currently, these shortcomings fall on the health of the consumer.

## Data Availability

Data information can be provided by the corresponding author upon request.

## References

[1] Scallan M. (2015). Ancient Ink: Iceman Otzi Has the World’s Oldest Tattoos. *Smithsonian Institution*.

[2] Jackson C. (2019). More Americans Have Tattoos Today Than Years Ago. *Ipsos*.

[3] Kluger N. (2017). Cutaneous Complications Related to Tattoos: 31 Cases from Finland. *Dermatology*.

[4] Warshaw E. M., Schlarbaum J. P., Taylor J. S. (2020). Allergic Reactions to Tattoos: Retrospective Analysis of North American Contact Dermatitis Group Data, 2001-2016. *Journal of the American Academy of Dermatology*.

[5] DermNet (2019). Tattoo-Associated Skin Reactions. https://dermnetnz.org/topics/tattoo-associated-skin-reactions#:~:text=The%20two%20most%20common%20hypersensitivity.

[6] Goldbio (2023). Safety Data Sheet True Black SDS.

[7] Goldbio (2023). Safety Data Sheet Bowery Red SDS.

[8] Goldbio (2023). Safety Data Sheet Bowery White SDS.

[9] Granlund H. (1994). Contact Allergy to Witch Hazel. *Contact Dermatitis*.

[10] Suzuki R., Fukuyama K., Miyazaki Y. (2016). Namiki T.Contact Urticaria Syndrome and Protein Contact Dermatitis Caused by Glycerin Enema. *JAAD Case Reports*.

[11] García-Gavín J., Lissens R., Timmermans A., Goossens A. (2011). Allergic Contact Dermatitis Caused by Isopropyl Alcohol: A Missed Allergen?. *Contact Dermatitis*.

[12] Weimann S., Skudlik C., John S. M. (2010). Allergic Contact Dermatitis Caused by the Blue Pigment VINAMON® Blue BX FW—A Phthalocyanine Blue in a Vinyl Glove. *JDDG: Journal der Deutschen Dermatologischen Gesellschaft*.

[13] Tsiogka A., Liopyris K., Gregoriou S. (2022). Allergic Contact Dermatitis Associated With Benzophenone Sensitization after a Recent Black Ink Tattoo. *Contact Dermatitis*.

[14] Ogura Y., Morimoto H., Otsuka M., Tokura Y. (2021). Paraphenylenediamine Ingredient Possibly Contributes to Granuloma Formation in Inflammatory Tattoo. *Journal of Cutaneous Immunology and Allergy*.

[15] DeLeo V. A. (2023). p-Phenylenediamine. *Dermatitis: Contact, Atopic, Occupational, Drug*.

[16] Macneil B., Saladof J. (2006). Heena Tattoo Ingredient is Allergen of the Year. *Skin and Allergy News*.

[17] de Groot A. C. (2013). Side-Effects of Henna and Semi-Permanent, Black Henna, Tattoos: A Full Review. *Contact Dermatitis*.

[18] Reck Atwater A. (2020). Tattoo Hypersensitivity Reactions: Inky Business. *Cutis*.

[19] Serup J., Hutton Carlsen K., Dommershausen N. (2019). Identification of Pigments Related to Allergic Tattoo Reactions in 104 human Skin Biopsies. *Contact Dermatitis*.

[20] FDA (2022). FDA Advises Consumers, Tattoo Artists, and Retailers to Avoid Using or Selling Certain Tattoo Inks Contaminated With Microorganisms. https://www.fda.gov/cosmetics/cosmetics-recalls-alerts/fda-advises-consumers-tattoo-artists-and-retailers-avoid-using-or-selling-certain-tattoo-inks.

